# Health Versus Disease as the Catalyst for Biomedical Research: The Science of Adipokines as a Case in Point

**DOI:** 10.3389/fendo.2014.00136

**Published:** 2014-08-15

**Authors:** Giamila Fantuzzi

**Affiliations:** ^1^Department of Kinesiology and Nutrition, University of Illinois at Chicago, Chicago, IL, USA

**Keywords:** leptin, adiponectin, adipokines, history, 20th century, pathology, physiology

## Catalysts for Biomedical Research

A common piece of advice when mentoring biomedical researchers preparing their first grant proposals is to focus on a specific disease or narrow range of conditions. The assumption is that proposing to investigate the pathological aspects of a problem – to study deviation from homeostasis – makes for a stronger project than approaching research through the lens of physiology, the study of the homeostatic state. Using the discovery of the adipokines leptin and adiponectin as an example, I question this assumption and discuss how both approaches can generate excellent research.

## Leptin and Adiponectin: Beginning with Pathology Versus Physiology

Adipokines are messenger proteins produced by adipocytes; they regulate a variety of functions from appetite to metabolism, inflammation, reproduction, and more (1). Leptin and adiponectin are the two best-known adipokines: leptin mostly regulates appetite, though it also profoundly affects metabolism, reproduction, and immunity, whereas adiponectin is for the most part involved in modulation of metabolic responses and inflammation (2).

Both discovered in the mid-1990s, leptin and adiponectin were initially identified through conceptually distinct research approaches (3–7). In fact, while the discovery of leptin can be thought of as part of the process of investigation into the pathological causes of obesity, adiponectin was instead isolated *via* experiments whose purpose can best be described as aimed at improving our understanding of basic adipocyte biology.

Comparing sentences from the original articles reporting the discovery of these two adipokines can help better clarify this point. In the introduction to their 1994 Nature report describing the identification of leptin, Zhang and colleagues wrote: “*Obesity is the commonest nutritional disorder in Western societies*” and “*The molecular pathogenesis of obesity is unknown*” (7). The stated impetus for research here is clearly a pathological condition, obesity, and the aim is “*the cloning and sequencing of the mouse obesity gene and its human homologue*.” (7). These concepts can be contrasted with those presented in the four articles describing the discovery of adiponectin that appeared in 1995–1996 (3–6). Here the authors used very different words to introduce their findings: “*Adipocytes secrete several proteins potentially important in homeostatic control of glucose and lipid metabolism*” (6); “*Increasing evidence [*…*] suggests that adipose tissue produces and secretes several proteins [*…*] thought to regulate adipose tissue function and to affect the metabolism of the whole body*” (4); “*Molecules secreted from adipose tissue are capable of modulating diverse functions in fat and other tissues, thus representing a new facet of adipose tissue physiology*” (3); “*We assumed that there might be plasma proteins that bind to collagen fibers [*…*] and searched for such novel plasma proteins*” (5). As these quotes illustrate, each of the research teams that independently discovered adiponectin framed their investigation in terms of searching for potential factors that might influence homeostasis and regulate physiological functions.

Statements from the discussion sections of the two groups of articles are also illuminating. Compare this sentence from the leptin paper, “*Identification of ob now offers an entry point into the pathways that regulate adiposity and body weight and should provide a fuller understanding of the pathogenesis of obesity*” (7), to words from the adiponectin reports, “*We do not yet know the function of Acrp30*” (6), “*The role of the apM1 protein is not clear at present*” (4), “*The identification of adipo-Q [*…*] poses many questions regarding its molecular and biochemical properties that are yet to be examined*” (3). (The original names for the leptin gene – *ob* – and for adiponectin – Acrp30, adipo-Q, and apM1 – are used in these sentences.) While the leptin paper presents the discovery as leading to a better understanding of a specific pathology, each of the adiponectin reports leaves the doors open as to the potential meaning of their findings. Thus, even though studies leading to the discovery of leptin and adiponectin can both be classified as basic research, the difference in approach and conclusions could not be clearer: understanding a pathological state in the case of leptin versus clarifying the normal biology of cells and tissues for adiponectin. Moreover, the discovery of leptin can be framed as identification of the gene long known to be involved in obesity, therefore falling into the category of specific hypothesis-driven research. In contrast, each of the projects that led to the discovery of adiponectin could easily be criticized for being a “fishing expedition.”

## Developments of Research on Leptin and Adiponectin

Just a few months after cloning of the leptin gene, the effect of leptin injections in reducing the body weight of mice were reported by three different groups in the same issue of Science (8–10). A news report commenting on these findings discussed the acquisition of the license to develop leptin by the biotech company Amgen and the potential for turning the discovery into obesity drugs (11). Given the public health and financial issues at stake, Phase 1 and 2 studies were conducted with unusual swiftness, showing that leptin was safe, but also that its effectiveness in curbing appetite and reducing obesity was variable and less than stellar (12). Meanwhile, data began accumulating on the pathophysiological changes in leptin production and signaling that take place in obese subjects (13), eventually leading to realization of the unfeasibility and lack of strong biological basis for using leptin as a therapeutic approach to obesity. In the early-to-mid 2010s, a modified version of the protein, metreleptin, was approved in several countries as a therapy for generalized lipodystrophy, a rare condition characterized by extremely low levels of leptin. Injections of leptin are also the only available life-saving treatment for the handful of individuals with congenital leptin deficiency. Despite the relative disappointment of leptin as a blockbuster drug for obesity, the study of leptin’s biology has literally revolutionized the way we understand the physiology of appetite and metabolic regulation. The importance of leptin’s discovery for scientists working on these basic problems, and for people with those rare conditions for which leptin is effective, cannot be overstated.

Much like its discovery, the history of adiponectin’s research can also be construed as somewhat opposed to that of leptin. It took three full years before the initial hunch that obesity is associated with reduced adiponectin levels (3) was to be reexamined (14). There were only 34 primary research articles on adiponectin in the first 5 years after its discovery, as opposed to the more than 2000 on leptin, and it was not until 2001 that adiponectin made it to the pages of high-impact journals (15, 16). Research then proceeded swiftly, with publications increasing exponentially. A whole lot of excellent biology went into the characterization of adiponectin as the “good adipokine” that promotes healthy metabolism and controls inflammation (17). Adiponectin agonists that may in the not-too-distant future potentially be used for promoting metabolic health and perhaps treat diabetes and other obesity co-morbidities should clinical studies demonstrate efficacy were identified in 2013 (18). As much as leptin revolutionized the biology of appetite, the discovery and characterization of adiponectin has had a radical, far-reaching effect on our understanding of the regulation of whole-body metabolic pathways. The concept of healthy cells producing proteins that act as messengers of health, as contrasted to diseased cells generating messages of distress, has had meaningful impact not only for the field of obesity and metabolism but as an overall framework to conceptualize the relationship between health and disease (19).

## The Stories We Tell

The history of the discovery of leptin and adiponectin can be used as a case in point to illustrate the importance of the logical frameworks used to justify biomedical research. Although it is well possible that the discoverers of leptin were actually more interested in the basic biology of appetite than in contributing to understand the pathophysiology of obesity, they chose to construe their findings in light of disease rather than health, of pathology instead of physiology. In contrast, the discovery of adiponectin was portrayed as an effort to understand the basic physiology of adipose tissue and collagen-binding proteins, with only passing reference to the potential medical implications of the findings.

Historians and philosophers of science tell researchers that what goes into scientific reports is not to be trusted, obviously referring not to the data presented but rather to the story being told, to the narrative used to explain and justify the purpose and meaning of a particular study. Nobel Prize winner Peter Medawar wrote: “*It is no use looking to scientific papers, for they not merely conceal but actively misrepresent the reasoning that goes into the work they describe*” (20), calling the outcome “*the studied hypocrisy expected of a contribution to a learned journal*” (21). The same concept can easily be applied to the writing of research proposals, which need to construe a credible, logical train of thought for the critical eye of the reviewer. But here indeed lies the crux of the matter, since the way we decide to narrate our “studied hypocrisy” is telling of the way scientists and, as a consequence, the general public perceives what is to be valued and important and what is not. By choosing to tell our stories in terms of disease rather than health, of pathology versus physiology, are we favoring a view that sees health as a passive state? Our bodies spend an incredible amount of energy (meant here in its physical form of calories and ATP, not as a metaphor) to actively promote health. To name just a few, adipocytes produce and release tremendous amounts of adiponectin – sufficient to maintain plasma levels in the tens of micrograms per milliliter range – that causally contribute to metabolic homeostasis; endothelial cells fine-tune production of nitric oxide to maintain appropriate vascular responsiveness; a complex network of signals allows for the presence of a functional pool of T regulatory cells that tame immune responses; sustained levels of autophagy recycle damaged and dysfunctional cellular components. Yet, these health-promoting mediators and responses are more often than not studied in the context of disease, not health. It is perhaps only a subtle change of perspective, but telling the story as originating from disease rather than health perpetuates a view that sees health as merely the absence of disease.

We should therefore ask ourselves whether we are using the right approach by emphasizing pathology over physiology as the rationale for important biomedical research, a choice that may lead to discount and ignore projects with potential for significant impact while contributing to perpetuate an outdated concept of passive health and static physiology. As exemplified by the history of adiponectin and leptin, conceptualizing research as answering to either health or disease has equal chances to produce outstanding science.

## Conflict of Interest Statement

The author declares that the research was conducted in the absence of any commercial or financial relationships that could be construed as a potential conflict of interest.
